# Light-efficient channel attention in convolutional neural networks for tic recognition in the children with tic disorders

**DOI:** 10.3389/fncom.2022.1047954

**Published:** 2022-11-04

**Authors:** Fudi Geng, Qiang Ding, Wanyu Wu, Xiangyang Wang, Yanping Li, Jinhua Sun, Rui Wang

**Affiliations:** ^1^School of Communication and Information Engineering, Shanghai University, Shanghai, China; ^2^Department of Psychological Medicine, Children’s Hospital of Fudan University, National Children’s Medical Center, Shanghai, China

**Keywords:** tic disorder datasets, tic recognition, convolutional neural networks (CNN), LCA module, light-efficient channel attention

## Abstract

Tic is a combination of a series of static facial and limb movements over a certain period in some children. However, due to the scarcity of tic disorder (TD) datasets, the existing work on tic recognition using deep learning does not work well. It is that spatial complexity and time-domain variability directly affect the accuracy of tic recognition. How to extract effective visual information for temporal and spatial expression and classification of tic movement is the key of tic recognition. We designed the slow-fast and light-efficient channel attention network (SFLCA-Net) to identify tic action. The whole network adopted two fast and slow branch subnetworks, and light-efficient channel attention (LCA) module, which was designed to solve the problem of insufficient complementarity of spatial-temporal channel information. The SFLCA-Net is verified on our TD dataset and the experimental results demonstrate the effectiveness of our method.

## Introduction

Tic disorders (TD) are neurodevelopmental disorders characterized by recurrent motor and/or vocal tics, which usually occurs in childhood. Tics can be transient, as represented by the diagnosis Provisional Tic Disorder (PTD) in the 5th edition of the Diagnostic and Statistical Manual of Mental Disorders (DSM-5), or they can persist for over a year as a chronic condition, described in the DSM-5 as Tourette’s Disorder [also named as Tourette syndrome (TS)] and Persistent (Chronic) Motor or Vocal Tic Disorder (CTD) (American Psychiatric Association, 2013).

The latest epidemiological survey of mental disorders among school-age children and adolescents aged 6–16 in China mainland shows that tic disorder ranks fourth, with a time point prevalence rate of 2.5%, and the time point prevalence rates of the three common subtypes of PTD, CTD, TS are 1.2, 0.9, and 0.4%, respectively (Li et al., 2022).

Tics usually occur in bouts, and tic symptoms often wax and wane during the disease course. The tics in one individual can change from one form to another, and new forms of tics could emerge during the disease course, but usually manifest as some specific stereotype during a particular time period (Byler et al., 2015). The diagnosis of tics requires a series of complex processes including history collection, clinical examination and evaluation, auxiliary examinations, etc., for diagnosis and differential diagnosis. This work is undoubtedly time-consuming, manpower consuming and also needs more professionals especially more pediatric psychiatrists. However, presently child psychiatry is still a discipline in its nascent stage in China with less than 500 available qualified pediatric psychiatrists, and most of them practice in big cities, such as Beijing, Shanghai, Guangzhou, and Wuhan (Zheng and Zheng, 2015). In China, children with TD are often mainly under the care of general pediatricians and pediatric neurologists. Pediatric psychiatrists are usually consulted for or in charge of more severe cases with mental comorbidities.

The early recognition rate of tic disorder from children and adolescents in current China mainland is still relatively low. Many patients are considered by the public and even some primary care doctors that tic symptoms are just bad habits and minor defects, which do not need special treatment. Some patients, especially TS and CTD patients, have not been diagnosed in time and standardized treatment. It is an important work that how to find and diagnose tic disorder early, especially in the general population in schools, and carry out large-scale screening, which can make up for the current shortage of professional personnel such as child psychiatrists in Chinese Mainland. Undoubtedly, artificial intelligence can play a role in assisting diagnosis and discovering TD patients in advance. However, at present, there are few literatures (Wu et al., 2021) on intelligent diagnosis of tic disorder in China Mainland.

Recently, artificial intelligence and machine learning have been widely applied in the medical field. But only a few studies have been published regarding the automatic detection of TS-related motor disturbances. The existing assessment scale has been simplified into qualitative and quantitative assessments of movements and sound twitches over a certain period, but it must still be completed manually. Rijn (2014) extracts the significant features such as eye blinking and uses support vector machine to automatically detect tic motion. Cernera et al. (2022) record 17 TS patients and calculate the spectral characteristics of the captured tic motion, and use it as the feature of support vector machine to detect tic motion.

Deep learning technology has developed rapidly, and been widely used in various classic visual tasks including but not limited to: image classification (Kim et al., 2020), object detection (Fan et al., 2020), and pose estimation (Xiao et al., 2018; Sun et al., 2019; Xiangyang Wang et al., 2021). It turns out that deep learning can eliminate the need for artificial feature extraction. Barua et al. (2021) propose a deep learning approach for detecting TD using wireless channel information and achieved an accuracy above 97%. The data used in the task are simulated using healthy human subjects. Chappell et al. (1994) show that, in addition to a severity scale, the severity of tic disorder can also be determined by recording the patient’s tics with video for more than 10 min. Moreover, monitoring and recording patients in their natural states instead of facing a clinician effectively avoids interference in diagnosis and evaluation caused by the patient actively controlling their tics. Based on real clinical data, Wu et al. (2021) propose a deep learning architecture that combines both unsupervised and supervised learning methods and learns features from videos for tic motion detection.

However, the number of datasets related to tic recognition is scarce. Deep learning networks require large amounts of labeled data, supervised learning improves the accuracy of deep learning network model directly. For video action recognition, there is no suitable network for recognizing fine movements such as tic motion. Slow-fast network (Feichtenhofer et al., 2019) adopts two fast and slow subnetwork, which are respectively, used to identify the actions of relatively static parts and fast movements in video. Although it has good recognition effect, the information between temporal channel and spatial channel is not well fused in this network. Some fine tics will be incorrectly recognized.

**FIGURE 1:**
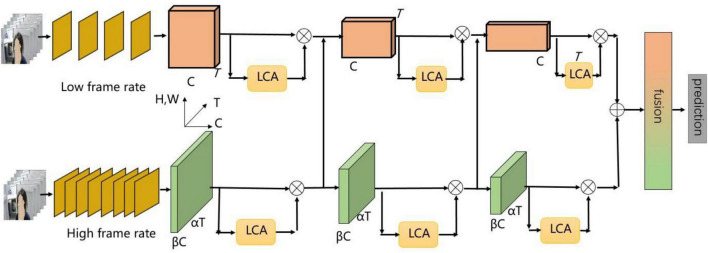
The overall architecture of slow-fast and light-efficient channel attention network for tic recognition.

From these perspectives, we build the TD dataset for the training of our tic recognition. And we propose slow-fast and light-efficient channel attention network (SFLCA-Net) for tic recognition. The network architecture is shown in Figure 1. We design two fast and slow subnetworks branches to extract spatial information and temporal information, respectively. Then, we design a module to improve the network structure for each part of the output feature maps, which is named light-efficient channel attention (LCA), in order to solve the problem of insufficient information complementarity between temporal channels and space channels. We verify the effectiveness of our proposed SFLCA-Net on our TD dataset and achieve high performance and excellent recognition effect.

Our main contributions are summarized as follows:

• We design a two branch subnetwork to capture the characteristics of fine tic movements, and propose an attention module to improve network structure for each part of the output feature maps, named SFLCA-Net, to solve the lack of complementarity of information between temporal channels and spatial channels.
• We build and utilize our TD dataset to train our tic recognition network, and achieve high performance and excellent recognition effect.

## Related work

### Double flow and 3D-convolution network

Simonyan and Zisserman (2014) propose that RGB images and optical flow images are sent into two networks and fused, so that the action information of objects in the video can be obtained. Xu et al. (2019) propose a framework based on the dense expansion network, and discussed different fusion methods of spatial and temporal branches. Due to the good effect of ResNet in image recognition tasks, Tran et al. (2015) propose using 3D neural network (C3D) instead of 2D neural network in video action recognition. Deep mind team (Carreira and Zisserman, 2017) proposes inflated 3D ConvNets (I3D), uses 2D network weight expansion as the pretraining weight of 3D network, and pretrains the network with the help of large-scale dynamics dataset. Combining the merit of double flow and 3D convolution network, slow-fast network (Feichtenhofer et al., 2019) adopts two fast and slow subnetwork, which are respectively, used to identify the actions of relatively static parts and fast movements in video.

### Action recognition datasets

The UCF-101 (Soomro and Zamir, 2014) is one of the most famous datasets for action recognition. UCF-101 gives the largest diversity in terms of actions and with the presence of large variations in camera motion, object appearance and pose, object scale, viewpoint, complex background, etc. Kuehne et al. (2011) introduce the HMDB51 dataset, which contains a total of about 6,849 video clips distributed in a large set of 51 actions include laughing, talking, eating drinking, etc., and each class contains at least 101 video clips. More recently, The AVA dataset (Li et al., 2020) proposes an influential instance of a video task that is expensive to annotate, instead of a single label per video, single person in a subset of frames gets a set of labels. These datasets greatly boost the progress of research on action generation. However, the current datasets about TD are quite limited in at least one respect: the number of categories, the samples of each category, the time for each sample, the diversity of video capture environment. In order to further improve the accuracy of tic recognition, it is necessary to construct relevant TD datasets.

### The channel attention mechanism

The channel attention mechanism has shown great potential in improving the performance of deep convolutional neural networks (CNN). The representative method is Squeeze-Excitation Network (SENet) (Hu et al., 2018). It is proposed to achieve an effective channel attention mechanism for the first time. It mainly learns channel attention weights of each convolution block and brings significant performance gain for various deep CNN architectures. Subsequently, the development of attention mechanisms is mainly divided into two research directions: enhanced feature aggregation and the combination of channel and spatial attention. Convolutional block attention module (CBAM) (Woo et al., 2018) adopts both Average-pooling and Max-pooling for feature processing, and a 2D convolution of k × K kernel size is employed to calculate the spatial attention map, then combines it with the channel attention map. GCNet (Cao et al., 2019) simplifies the Non-Local Network (Wang et al., 2018) and integrates with SE blocks, leading to the lightweight module remote dependency model.

## Slow-fast and light-efficient channel attention network for tic recognition

### Overall network structure

The slow-fast network (Feichtenhofer et al., 2019) structure is introduced to design two fast and slow subnetworks branches. We design two-stream convolution input, a slow channel and a fast channel, to extract spatial information and temporal information, respectively. For fast subnetwork branch, we use a larger number of frames and fewer channels to learn tic motion information, and for slow subnetwork branch, we use a smaller number of frames and a larger number of channels to learn spatial semantic information. During every stage, the features extracted from the fast subnetwork branch flows to the slow subnetwork branch, and _1×1_ convolution is used to fuse the temporal and spatial semantic information of different channels. In the last stage, the fast and slow subnetworks branches are fused, and then are input into the full connection classification layer.

**FIGURE 2:**
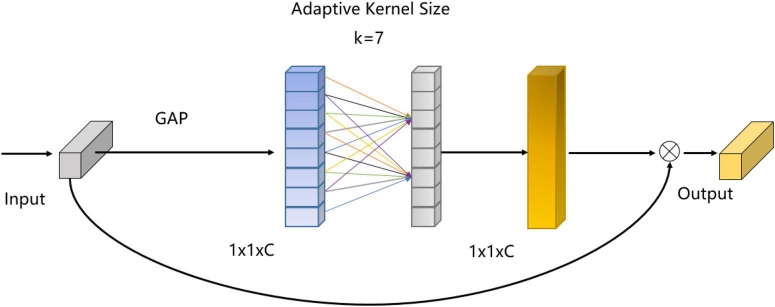
Light efficient channel attention module.

The network adopts the backbone network of 3D-ResNet (Hara et al., 2017). For the backbone network, the feature channel does not interact well before the last full connection classification layer. It leads to the lack of complementarity of information between temporal channels and spatial channels. The network lacks detailed spatial-temporal feature extraction in each branch, and the subtle change characteristics of tic movements are easy to be ignored. Therefore, in order to solve this problem, as shown in Figure 2, we design LCA module. For each part of the output feature maps, _1×1_ convolution is used to capture the cross-channel interaction information. The effectiveness and robustness of the SFLCA-Net network for tic recognition are verified.

### Light-efficient channel attention module

In order to improve the identification performance of tic recognition network and overcome the contradiction between performance and complexity, we design the LCA module, which involves only a few parameters but with obvious performance. This design avoids dimensionality reduction, which is an effective strategy for learning channel attention maps. And it appropriates cross-channel interaction can significantly reduce model complexity while maintaining performance. The LCA module captures local cross-channel interactions by considering each channel and K adjacent channels. In addition, the LCA module adaptively selects the size of the convolution kernel to determine the coverage of local cross-channel interactions. When the number of input channel of is less than 7, the size of the convolution kernel decreases in proportion to the number of input channel. It can prevent the receptive field from being larger than the size of the feature map due to the large size of convolution kernel. When the number of input channel of is larger than 7, the convolution kernel size is kept at 7 to reduce the parameters of the light-efficient channel attention module. We insert the LCA module into our backbone and propose the SFLCA-Net for tic recognition.

GAP represents global average pooling, ⊗ denotes the element-wise multiplication. The receptive field of adaptive kernel size is k × k. The LCA module is formulated as follows:


(1) $$F_{c}=S\,i\,g\,m\,o\,i\,d\,\left(W_{1\,d}\,\left(G\,A\,P\,\left(X\right)\right)\right)$$

The LCA module need to compress the input feature X ∈ ℝ^*C*×*H*×*W*×*T*^ in the spatial dimension for effectively calculating the channel attention map. The result of the 3D global average pooling of X ∈ ℝ^*C*×*H*×*W*×*T*^ is as follows:


(2) $$F_{1}=G\,A\,P\,\left(X\right),F_{1}\in {\mathbb{R}}^{C\times 1\times 1\times 1}$$

(3) $$F_{2}=R\,e\,L\,U\,\left(W_{1\,d}\,\left(F_{1}\right)\right),F_{2}\in {\mathbb{R}}^{C\times 1\times 1\times 1}$$

(4) $$F_{c}=\text{X}⊗S\,i\,g\,m\,o\,i\,d\,\left(F_{2}\right),F_{c}\in {\mathbb{R}}^{C\times 1\times 1\times 1}$$

Then 1D convolution layer is utilized to model the channel relationships of *F*_1_ ∈ ℝ^*C*×1×1×1^ without the reduction of the channel dimension. In addition, the kernel size of *W*_*1d*_ is adaptive to avoid superabundant parameters in the network. Finally, the sigmoid function is employed to activate the *F*_2_ ∈ ℝ^*C*×1×1×1^ as a channel attention weight map, and then multiplied with the input feature X ∈ ℝ^*C*×*H*×*W*×*T*^ to produce the output feature. After a series of procedures described above, the LCA module can realize efficient cross-channel interaction.

## Experiments

### Datasets of tic disorder

The TD dataset is focused on childhood-onset tic recognition and contains 13 categories such as eye blinking, mouth twitching, head jerking, etc. Sixty-eight children (4–13 years old) diagnosed with TD by an experienced psychiatry specialist according to the criteria for tic disorder from DSM-5 (American Psychiatric Association, 2013). All participants were outpatients under routine treatment recruited from the department of psychological medicine, affiliated pediatric hospital of Fudan University. The study received approval from the local ethics committee. Full written informed consent was obtained from each participant’s parents before this study. A written commitment was signed by research group to protect the privacy of patients and respect the right to portrait of patients. The researchers attend this study all guarantee that the identifiable information of patients cannot be leaked to the public network and is only used for this study.

Our dataset has made significant progress in terms of the number and variety of parallel actions, as well as video resolution and total frames for tic recognition. Some features of our dataset are compared to human action detection datasets in Table 1. The TD dataset is based on more than 80 real-world TD patients. The dataset has 20–1,000 clips for each category and each clip lasts around 5 s. The current version includes 2,234 videos, and is divided into three subsets, one for training set with 1,566 videos, one for validation set with 443 videos, and one for test set with 225 videos.

**TABLE 1:** Comparison of Tic disorder dataset with current fully annotated human action detection datasets.

Dataset	Number of actions	Total frames	Average video seconds	Resolution	Resource
UT-Interaction Ryoo and Aggarwal, 2009	6	36K	60 s	720 × 480	Actor staged
UCF-101 Soomro and Zamir, 2014	24	558K	5.8 s	320 × 240	YouTube
J-HMDB Kuehne et al., 2011	21	32K	1.4 s	320 × 240	YouTube
LIRIS-HARL Wolf et al., 2014	10	64K	15.2 s	720 × 576	Actor staged
Okutama-Action Barekatain et al., 2017	12	77K	60 s	3840 × 2160	Actor staged
**TS-Data (ours)**	13	412K	4 s	1920 × 1080	TS patients

### Implementation details

We train our network on videos using standard SGD with momentum in all cases with synchronous parallelization across four 3090 NVIDIA GPUs for all models. We train models with the tenfold reduction of learning rate when validation loss saturated, and tune weight decay and learning rate hyperparameters on the validation set of our TD dataset. All the models are implemented in PyTorch.

The original clips have variable resolution and frame rate. In our experiments, they are all normalized so that the larger image side is 128 pixels wide for our models. We also resample the videos so they have 120 frames for each video clips. In addition, data augmentation is known to be of crucial importance for the performance of deep architectures. We use random cropping both spatially–randomly cropping a 256 × 256 patch and temporally–randomly cropping a 90 frames cube, when picking the starting frame among those early enough to guarantee a desired number of frames. For shorter videos, we loope the video as many times as necessary to satisfy each model’s input interface. Random left-right flipping consistently is applied for each video during training.

Due to the small number of the samples in TD dataset, it’s necessary to increase the size of the training set appropriately to achieve a better training network effect. Therefore, the training set, validation set and test set are divided into 8:1:1. And the top-1 and top-5 classification accuracy (%) (Simonyan and Andrew, 2014) is introduced for experiments validation. Top-1 classification accuracy refers to the proportion of the number of correct labels in all test images that are the best classification probability in all test images. Top-5 classification accuracy refers to the proportion of the correct labels in all test images that are included in the first five classification probabilities in all test images.

### Main results and analysis

#### Analysis of the influence of different backbones

In this experiment, we study the performance of different backbone networks on the dataset. The backbone networks used are set as 3D-ResNet-18, 3D-ResNet-34, and 3D-ResNet-50, respectively. The experimental results are shown in Table 2, the top-1 score achieves 88.89% and the top-5 score achieves 99.56% when adopt the backbone of 3D-ResNet-18. The top-1% score achieves 89.33% and the top-5 score achieves 99.56% when the backbone is set to 3D-ResNet-34. The top-1% score achieves 84.00% and the top-5 score achieves 100% on the backbone of 3D-ResNet-50.

**TABLE 2:** Results of different backbones on the Tic disorder dataset.

Backbone	Top-1%	Top-5%
3D-ResNet-18	88.89	99.56
3D-ResNet-34	89.33	99.56
3D-ResNet-50	84.00	100

According to the above experimental results, the accuracy has achieved good results when the backbone network is set to 3D-ResNet-18. The efficiency of the model prediction results is guaranteed. When the backbone network is set to 3D-ResNet-34, only the top-1% result is improved. In contrast, the score is only increased by 0.44. The improvement effect of the network model is not great, but the deepening of the model affects the real-time effect. When the backbone network is set to 3D-ResNet-50, the result of top-1% decreases by 4.89%, but the score of top-5 reaches 100%. But for the task of childhood-onset tic recognition, top-1 is more important. According to the experimental analysis, 3D-ResNet-18 is more robust for tic action recognition on this dataset.

#### Influence of input video frame rate

In this experiment, we study the impact of different sampling rates of childhood-onset tic videos on the performance of the model. The four sampling rates are set to 1, 2, 3, and 5, respectively. The experimental results are shown in Table 3, when the sampling rate is set to 1, the top-1% score reaches 88.89% and the top-5 score reaches 99.56%. When the sampling rate is set to 2, the top-1% score reaches 88.57% and the top-5 score reaches 98.66%. When the sampling rate is set to 5, the top-1% score reaches 96.40%, and the top-5 score reaches 97.64%.

**TABLE 3:** Results of different sampling frame rate on the Tic disorder dataset.

Backbone	Sampling frame rate	Top-1%	Top-5%
3D-ResNet-18	1	88.89	99.56
3D-ResNet-18	2	88.57	98.66
3D-ResNet-18	3	85.78	98.22
3D-ResNet-18	5	86.40	97.64

According to the above experimental results, the performance of the model decreases gradually with the increase of the video sampling rate. When the sampling rate exceeds to 3, the robustness of the model decreases sharply. It can be seen that the information contained in continuous frames can deal with subtle changes in childhood-onset tic movements. When the frame sampling rate is too large, the continuity between twitching frame images decreases, and the slightly changed information is lost, resulting in misjudgment of the model and reduced robustness. In other words, different from general motion recognition, for childhood-onset tic recognition, the input of continuous frames is very important for the effect of the model.

#### Analysis of the light-efficient channel attention module

In this experiment, we study the impact of childhood-onset tic recognition on the SFLCA-Net network. We first select 3D-ResNet-18 as the backbone network and add the LCA module to the block in the network. The experimental results are shown in Table 4, when the LCA module is added, the score of top-1 reaches 92.00%, and the accuracy is improved by 3.11. The top-5 score reaches 100%.

**TABLE 4:** Results of light-efficient channel attention (LCA) model on the Tic disorder dataset.

Method	Backbone	Top-1%	Top-5%
Slowfast Wang et al., 2021	3D-ResNet-18	88.89	99.56
SFLCA-Net	3D-ResNet-18	92.00	100

The experimental results are better than using backbone 3D-ResNet-34 and 3D-ResNet-50 by adding the LCA module. The fusion of spatiotemporal information is important for childhood-onset tic symptoms recognition to ensure continuity in the time domain. LCA module can solve the problem of lack of information complementarity between temporal channel and spatial channel of Slow-fast network, and solve the lack of detailed spatial-temporal feature in each branch. It cannot only ensure the accuracy of model prediction and improve the robustness of the model, but also improve the real-time effect of the model.

## Discussion

Tic disorders are neurodevelopmental disorders characterized by recurrent motor and/or vocal tics, which usually occur in childhood. During the long-term observation, diagnosis of TD children, tic motion need to be continuously monitored and recorded. Reviewing and evaluating these monitoring data is a time and cost-effective process for doctors. However, application of artificial intelligence can save time and cost, enable doctors to optimize and adjust drug response, and help establish a good evaluation and management process for patients. Our work is to construct the tic disorder dataset, and use the video recognition model to recognize the tic motion through deep learning.

As shown in the experiment, we first use the video based tic recognition model, which has been able to preliminarily recognize multiple different tic motion subjects. However, in order to apply it to medical diagnosis, we still need to improve the accuracy. Therefore, combined with the advantages of the existing models and the characteristics of tic motion, we propose a relatively lightweight model, which cannot only improve the accuracy, but also ensure the efficiency of tic recognition automatically. Through the experiment, the recognition accuracy of our SFLCA-Net has reached 92.0%. However, the TD dataset has only action video and no audio data. Therefore, we need to expand the TD dataset to audio data in order to recognize tic motion more comprehensively in the future.

From our perspective, this work has the following application prospects: One is to use the TD dataset to recognize the tic motion through deep learning, give the preliminary diagnosis results, and improve the work efficiency of doctors; The second is to train the artificial intelligence system by collecting a large number of tic video data and clinical diagnosis information, so as to make it have independent diagnosis ability, reduce the misdiagnosis rate of complex diseases and improve the diagnosis level.

## Conclusion

For tic recognition, we need to constantly construct a reasonable and abundant TD dataset. Meanwhile, we need to design a network to match this kind of action. Extracting effective visual information for temporal and spatial expression and classification of tic movements is the key to the research of tic recognition. We designed SFLCA-Net network to recognize tics. The whole network adopts two fast and slow branch sub networks. LCA module is designed to solve the problem of insufficient complementarity of spatial-temporal channel information. The ablation study shows that employing the LCA module in our model can improve the performance of the baseline. Extensive experimental results on TD dataset demonstrate the effectiveness of our method.

## Data availability statement

The raw data supporting the conclusions of this article will be made available by the authors, without undue reservation.

## Author contributions

FG performed the data analyses and wrote the manuscript. WW performed the experiment. XW contributed significantly to analysis. YL performed the data analyses. JS contributed to the conception of the study and recruited the subjects. RW helped perform the analysis with constructive discussions. QD contributed to the acquisition of data. All authors contributed to the article and approved the submitted version.
